# DNMT3a-Mediated Enterocyte Barrier Dysfunction Contributes to Ulcerative Colitis via Facilitating the Interaction of Enterocytes and B Cells

**DOI:** 10.1155/2022/4862763

**Published:** 2022-05-06

**Authors:** Bo Cheng, Ai-mei Rong, Wenlu Li, Xiuqian Bi, Xinguang Qiu

**Affiliations:** ^1^Department of Emergency Surgery, The First Affiliated Hospital of Zhengzhou University, Zhengzhou 450000, China; ^2^Department of Gastroenterology, Zhengzhou Central Hospital Affiliated to Zhengzhou University, Zhengzhou 450000, China; ^3^Department of Stomatology, The First Affiliated Hospital of Zhengzhou University, Zhengzhou 450000, China; ^4^Department of Thyroid Surgery, The First Affiliated Hospital of Zhengzhou University, Zhengzhou 450000, China

## Abstract

**Objective:**

Dysfunction of the enterocyte barrier is associated with the development of ulcerative colitis (UC). This study was aimed at exploring the effect of DNMT3a on enterocyte barrier function in the progression of UC and the underlying mechanism.

**Method:**

Mice were given 3.5% dextran sodium sulphate (DSS) in drinking water to induce colitis. The primary intestinal epithelial cells (IECs) were isolated and treated with lipopolysaccharide (LPS) to establish an *in vitro* inflammatory model. We detected mouse clinical symptoms, histopathological damage, enterocyte barrier function, B cell differentiation, DNA methylation level, and cytokine production. Subsequently, the effect of DNMT3a from IECs on B cell differentiation was explored by a cocultural experiment.

**Result:**

DSS treatment significantly reduced the body weight and colonic length, increased disease activity index (DAI), and aggravated histopathological damage. In addition, DSS treatment induced downregulation of tight junction (TJ) protein, anti-inflammatory cytokines (IL-10 and TGF-*β*), and the number of anti-inflammatory B cells (CD1d+) in intestinal epithelial tissues, while upregulated proinflammatory cytokines (IL-6 and TNF-*α*), proinflammatory B cells (CD138+), and DNA methylation level. Further *in vitro* results revealed that DNMT3a silencing or TNFSF13 overexpression in IECs partly abolished the result of LPS-induced epithelial barrier dysfunction, as well as abrogated the effect of IEC-regulated B cell differentiation, while si-TACI transfection reversed these effects. Moreover, DNMT3a silencing decreased TNFSF13 methylation level and induced CD1d+ B cell differentiation, and the si-TNFSF13 transfection reversed the trend of B cell differentiation but did not affect TNFSF13 methylation level.

**Conclusion:**

Our study suggests that DNMT3a induces enterocyte barrier dysfunction to aggravate UC progression via TNFSF13-mediated interaction of enterocyte and B cells.

## 1. Introduction

Ulcerative colitis (UC) is an inflammatory bowel disease that occurs mainly in the colonic mucosa [1, 2]. The main clinical manifestations of the patients are chronic diarrhea and mucous pus with blood stool [3]. Generally, the disease is prone to recurrence, which increases the risk of cancer in patients [4, 5]. UC occurs mainly on the rectum and sigmoid colon, and the severe cases even implicate the entire colon [6]. The immune abnormality has been considered as one of the major pathogenic factors of UC [7]. The oral anti-inflammatory drugs, such as aminosalicylic acid, are the main drugs in clinical treatment and have not achieved satisfactory curative effects [8]. Recently, although several progress have been made on pathogenesis through focusing on immune response and inflammation [9, 10], the exact regulating mechanism is still not fully illustrated. Therefore, it is particularly important to investigate the pathogenesis of UC to discover new therapeutic targets.

The intestinal epithelium is a physical barrier required for nutrient uptake, which plays a key role in homeostasis through innate immune [11]. Intestinal epithelial cells (IECs) absorb nutrients and response to pathogens, acting as a barrier to coordinate immune responses [12]. The connective tissue between adjacent IECs forms an intestinal barrier that prevents harmful substances or pathogens from entering the body. During UC, intestinal mucosa produces a large number of inflammatory cytokines, which in turn induces apoptosis of IEC cells and affects the level of tight junction protein (TJ), resulting in impaired intestinal mucosal barrier function [13, 14]. B cells and their subsets differentiate into plasma cells that participate in inflammatory response by releasing anti-inflammatory cytokines [15]. For example, regulatory B (Breg) cells secrete TGF-*β* and IL-10 to inhibit the development of inflammation and protect the intestinal barrier function [16]. Thus, the pathogenesis of UC is related to B cell differentiation, subgroup imbalance, and cytokine production, which needs to be further elucidated.

DNA methylation is one of the major epigenetic modifications in mammals and is closely associated with transcriptional repression [17, 18]. A comparative analysis of genome-wide DNA methylation sequencing result revealed that gene methylation levels were different between UC patients and healthy controls, suggesting that DNA methylation plays an important role in the diagnosis and treatment of UC [19]. Currently, it has been demonstrated that DNMT1, DNMT3a, and DNMT3b are the 3 confirmed genes encoding proteins with DNA methyltransferase activity in mammals [20]. DNMT3a is a novel DNA methyltransferase with a molecular weight of 130-kDa, which is highly conserved in vertebrates and establishes DNA methylation through unmethylated CpG sites [20–23]. TNFSF13 (tumor necrosis factor superfamily member 13) is a cell proliferation-inducing ligand that is essential for B cell development [24]. TNFSF13 can be produced by a variety of cells, such as T cells, dendritic cells, monocytes, macrophages, and IECs [25, 26]. Previous studies claimed that IECs could mediate B cell differentiation by releasing TNFSF13 and cytokines [27]. However, the role of DNMT3a and TNFSF13 in UC remains unclear.

According to the result of bioinformatic analysis, the number of B cells in UC tissues was augmented with the increased DNA methylation level, suggesting that it was closely related to the progression of UC. Based on the bioinformatics result and previous evidence, we explored the effect of DNMT3a in regulation of enterocyte barrier function and B cell differentiation in the progression of UC to better understand the regulatory network of the disease.

## 2. Material and Method

### 2.1. Murine Colitis Model Establishment

Male C57BL/6 mice, aged 6-8 weeks, weighing 18-22 g were purchased from the animal center of Zhengzhou University, and were caged in specific pathogen free (SPF) environment with normal free food and water. During the study, mice were kept at 23 ± 2°C with a 12 h light-dark cycle. Mice received 3.5% DSS (36-50 kD, MP Biomedicals, USA) in drinking water for continuous 7 days, followed by regular drinking water for consecutive 3 days. The mice in the sham group received regular drinking water for consecutive 10 days. The animal study was approved by the Animal Ethics Committee of Zhengzhou University.

### 2.2. Histological Analysis

Disease activity index (DAI) scores were detected daily for continuous 7 days to evaluate the damage of colitis. DAI containing the measurement of body weight, stool consistency, and stool bleeding. DAI = (body loss rate + stool consistency + stool bleeding)/3. Each score was evaluated as follows: weight loss (0, none; 1, 1-5%; 2, 6-10%; 3, 11-15%; 4, over 15%); stool scores: (0, normal; 1-2, loose stools; 3-4, diarrhea); stool bleeding (hemoccult test): (0, negative; 1, positive; 2, blood present in the stool; 3, blood visibly and blood clotting on the anus; 4, gross bleeding). After the mice were sacrificed, all the colons (from the cecum to proximal rectum) were immediately removed. A tweezer was used to remove the adipose tissue and lymph nodes around the colon. Then, the colon was photographed and the length was measured. The next step, the colons were rinsed with cold PBS buffer to eliminate the fecal matter, and a 1-2 cm bowel was taken at the same location in each group. The shearing colonic tissue was fixed and stained with hematoxylin-eosin (H&E) solution as previously described [28, 29], and pathological changes were determined under a microscope (Nikon, Tokyo, Japan).

### 2.3. Isolation of IECs and Culture

The IECs were isolated followed the previous description [30]. Mouse intestines were longitudinally dissected into 2-3 mm length after washing with cold normal saline. In brief, intestinal epithelial tissue was scraped on ice with a glass slide and the mucosal swab was resuspended in cold PBS. The epithelial materials were centrifuged at 150 × *g*, followed by washing twice with cold PBS to reduce blood cell contamination. The isolated IECs were cultured in DMEM (Gibco, USA) containing 10% fetal bovine serum (FBS, Gibco), 2.5% penicillin-streptomycin-fungizone, and 1% gentamicin (Thermo Fisher, USA) in 6-well plates (corning, USA) at 37°C with 5% CO_2_. Cells were then subcultured and passaged every three days, and the 3-4 passage cells were used for the following experiments.

### 2.4. Isolation and Culture of CD19+ B Cells

B cells were isolated from the spleen of wild-type mice. Briefly, the spleens were cleaned with PBS and mashed to prepare the single cell suspension, followed by filtration through a 70 *μ*m cell strainer (BD Biosciences). Next, CD19+ B cells were isolated by using magnetic labeled with biotin-conjugated CD19 antibody and antibiotin microbeads (Miltenyi Biotech, Germany) according to manufacturer's guidelines. The isolated CD19+ B cells were cultured in RPMI-DMEM containing 10% FBS and 1% penicillin/streptomycin in 6-well plates (corning, USA) at 37°C with 5% CO_2_. Cells were subcultured and passaged twice a week, and the third- or fourth-passage cells were used for the following assay.

### 2.5. Cell Transfection

IECs or CD19+ B cells were seeded in 12-well plates (corning, USA) at a density of 5 × 10^5^ cells/well with 1 mL complete cultural medium overnight. For the transfection, the si-DNMT3a or si-NC (negative control), pc-TNFSF13 (containing EGF label), or pc-NC (negative control, containing EGF label) were transfected or cotransfected into IECs by using lipofectamine RNAiMAX reagent (Invitrogen, USA). The protocols for transfection were referred to the manufacturer's instructions. For CD19+ B cell transfection, the si-TACI or its negative control si-NC was transfected into CD19+ B cells using the same reagent and method as IEC transfection. All the siRNAs and vectors were synthesized by Genepharma Co.,Ltd (Shanghai, China). After transfection for 24 h, LPS (1 *μ*g/mL) was added to the cultural medium of IECs and stimulated for 48 h. The cells and cell supernatants were collected for the following examination.

### 2.6. Coculture of IECs and CD19+ B Cells

To understand the effect of IECs on B-cell differentiation, CD19+ B cells were cocultured with IECs in a cocultural system (24-well Transwell plate with upper and lower champer, corning, USA) after finishing the specific transfections in IECs and CD19+ B cells. 200 *μ*L IECs at a density of 3 × 10^5^ cell/mL were placed in the upper chamber, and 500 *μ*L CD19+ B cells (3 × 10^5^ cell/mL) were cultured in the low chamber. Next, LPS (1 *μ*g/mL) was added to the cultural medium of IECs and stimulated for 48 h. The cocultured cells were incubated at 37°C with 5% CO_2._ Subsequently, the IEC cell supernatant, IECs, and CD19+ B cells were collected for further detections.

### 2.7. Quantitative Real-Time PCR

According to the instructions, total RNA of colonic tissues was extracted by using the Trizol Reagent (Invitrogen, USA). The cDNA was synthesized with the PrimeScript™ Reverse Transcription Kit (TakaRa), and real-time qPCR reactions were carried out using the SYBR Green PCR Kit (Sangon Biotech, Shanghai, China) on a ROCHE LightCycler® 480 System according to the instructions. The relative mRNA levels of genes were quantified using the 2 ^-*ΔΔ*Ct^ method. *β*-Actin was used as the internal control. The primer sequences for mRNA quantitation were listed in Supplementary Table 1.

### 2.8. Western Blotting

Total proteins from colon tissue and IECs were extracted with RIPA buffer. The lysates were centrifuged at 12,000 × *g* for 15 min, and the protein concentration was detected with a BCA protein kit (Bio-Rad, USA). Next, the proteins were separated by SDS-PAGE and transferred onto the polyvinylidene fluoride (PVDF) membrane. The membrane was blocked with 5% skim milk at room temperature for 2 h and incubated with the primary antibodies against anti-occludin (ab216327, abcam, UK), anti-ZO-1(ab96587, abcam), anti-DNMT3a (ab188470, abcam), anti-TNFSF13 (ab108206, abcam), anti-TCAI (ab239370, abcam), and anti-*β*-actin (ab8226, abcam) at 4°C overnight. Then, the PVDF membrane was incubated with the HRP-conjugated secondary antibody. Finally, the bands were visualized by enhanced chemiluminescence (ECL) kit and gel imaging system.

### 2.9. Immunofluorescence Analysis

The colon tissues and IECs were collected and fixed in 4% paraformaldehyde and then performed routinely dewaxing and hydration. Then, the immunofluorescent staining was performed. The sections were incubated with primary antibodies of antioccludin (ab216327, abcam), anti-ZO-1(ab221547, abcam), and anti-DNMT3a (ab188470, abcam) overnight at 4°C. After fully washed with PBST, the sections were incubated with Alexa Fluor 488/594-labeled secondary antibody at room temperature for 1 hour. The nuclei were stained with DAPI (1 *μ*g/mL, Beyotime, China). The images were obtained by a fluorescence microscopy (Nikon, Japan).

### 2.10. Flow Cytometry

B cells were isolated from the spleens of mice. After finishing various treatments, cells were collected and washed with cold PBS, and then performed fluorescence-activated cell sorter (FACS) analysis. Cells were stained with fluorescein isothiocyanate-conjugated anti-CD16/32 (BioLegend, USA) and incubated for 15 min at 4°C in the dark. For CD19+CD5+CD1d^hi^ B cells staining, the antibodies of anti-CD19, anti-CD5, and anti-CD1d were added and incubated for 30 min at 4°C in the dark. For CD138+CD19+ B cells staining, the antibodies of anti-CD19 and anti-CD138 were added and incubated for 30 min at 4°C in the dark. After washing at 1500r and 5 min twice, the stained cells were analyzed by flow cytometry (Beckman, CA). To identify the living rate of isolated IECs, the Propidium Iodide ReadyProbes Reagent (Life Technologies) was used to incubate with IECs for 15 min, and the fluorescence intensity was analyzed by flow cytometry [31]. To confirm the purity of IECs, cells were stained with anti-CD326 (E-AB-F1181UD, Elabscience, Wuhan, China) for 30 min and analyzed on flow cytometry (Beckman, CA) [32]. As for evaluating transfection's efficiency of EGF-labeled pcDNA-TNFSF13 or pcDNA, the fluorescence rate was measured on a flow cytometry (Beckman, CA).

### 2.11. Enzyme-Linked Immunosorbent Assay (ELISA)

The mouse serum samples and B cell supernatants were collected and used for ELISA detection. The concentrations of IL-6, IL-10, TNF-*α*, and TGF-*β* were evaluated using the corresponding ELISA kits (eBioscience, USA) according to the manufacturer's instructions. The absorbance was measured at 450 nm with a microplate ELISA reader.

### 2.12. Genome-Wide DNA Methylation Detection

The methylation of genomic DNA in intestinal epithelial tissues of mice in sham and DSS-induced colitis group was detected by using a Methylated DNA Quantification Kit (Fluorometric, Abcam, UK) following manufacturer's instructions. DNA in intestinal epithelial tissues was extracted using a Universal Genomic DNA Extraction Kit (TaKaRa, Beijing, China). Finally, the absorbance was measured and read with 450 nm within 15 min.

### 2.13. Methylation-Specific PCR (MSP) Analysis

The methylated DNA in intestinal epithelial tissues and IECs was detected by using EZ DNA Methylation-Gold™ Kit followed the standard protocols. TNFSF13 primer for methylated and unmethylated was synthesized by Genescript Co. Ltd. (Nanjing, China). The cycle parameters for MSP were as follows: 95°C for 5 min, followed by 20, 30, and 40 cycles, 95°C for 30 s, 50-60°C for 30 s, and 72°C for 30 s, and finally extended for 7 min at 72°C. PCR products were the performed 3% agarose gel electrophoresis and stained with 0.5 *μ*g/mL ethidium bromide. The optical density was evaluated with Quantity One 4.6.2 (Bio-Rad, USA).

### 2.14. Measurement of Transepithelial Electrical Resistance (TEER)

The layer integrity of IECs was assessed by TEER measurements using a Millicell-ERS 2 V-Ohmmeter (Millipore, USA) following previous descriptions [32, 33]. After transfection of si-DNMT3a or si-NC, IECs were resuspended into cell suspension and seeded into a Transwell plate at a density of 5 × 10^5/^mL, followed by LPS treatment. The cell suspension (0.3 mL, 1.5 × 10^5^ cells/well) was added to the apical chamber. The low chamber seeded with the B cells (5 × 10^5/^mL) with 600 mL medium. After incubation for 5 days, TEER values were obtained and calculated through the formula: TEER (*Ω* × cm^2^) = (Rsample–Rblank) × effective membrane area (cm^2^), (Rsample: experimental resistance; Rblank: blank resistance).

### 2.15. Statistical Analysis

All data were expressed as means ± SD. Statistical analyses were conducted by SPSS 17.0 (IBM, SPSS) and GraphPad Prism 6. The one-way ANOVA analysis was used to compare the difference between multiple groups, and Student's *t* test was used to analyze the significance between two groups. *p* value <0.05 was considered statistically significant.

## 3. Results

### 3.1. Clinical Symptoms and Inflammatory Cell Infiltration in Mice with DSS-Induced Colitis

**Figure 1:**
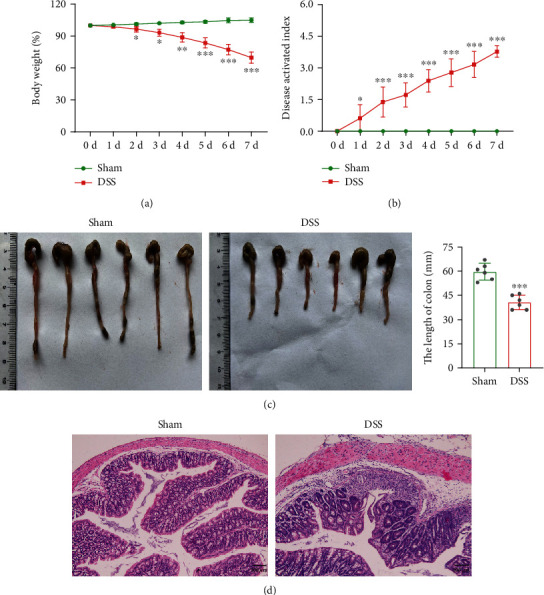
Clinical symptoms and inflammatory cell infiltration in mice with DSS-induced colitis. Mice were administered with 3.5% DSS to induce the model of colitis, and the sham group only received regular drinking water (*n* = 6 per group). (a) and (b) The body weight and DAI scores were evaluated daily. (c) Mice were sacrificed, and the colons (from the cecum to proximal rectum) were collected. Representative images of the colon samples were displayed, and the average lengths of colon were determined. (d) H&E staining was performed on proximal colonic tissues, and the representative images of colon pathologic abnormalities were exhibited. ^∗∗∗^*p* < 0.001 vs. sham group; scale bar = 100 *μ*m.

The DSS-induced mouse colitis model was established, and the clinical symptoms were observed. The bodyweight of mice was detected for 7 consecutive days, and the result showed that the bodyweight was gradually decreased in DSS group compared with the sham group (Figure 1(a)). In addition, a gradual increase of DAI was observed in DSS-induced mice (Figure 1(b)). Subsequently, the mice were euthanized and colon tissues were collected. As shown in Figure 1(c), the colon length was dramatically shortened in the DSS treatment group. Compared with the sham group, we discovered that DSS induced markedly pathological damage manifested with the neutrophil infiltration, crypt dilation, and goblet cell loss (Figure 1(d)).

### 3.2. DSS Treatment Induces Intestinal Epithelial Barrier Dysfunction and DNMT3a Level

**Figure 2:**
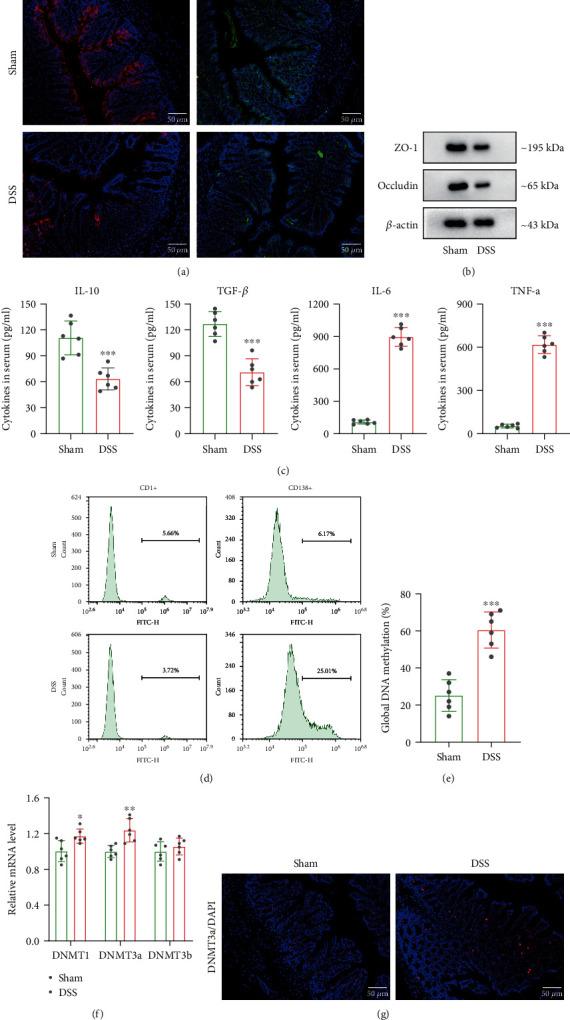
DSS treatment induces intestinal epithelial barrier dysfunction and DNMT3a level. The intestinal epithelial tissues of mice in the sham and DSS group were collected. (a) and (b) ZO-1 and occludin levels in intestinal epithelial tissues were measured by immunofluorescence staining and western blotting. (c) Levels of inflammatory cytokines containing L-10, TGF-*β*, IL-6 and TNF-*α* in serum of mice were evaluated by ELISA. (d) The number of CD1d+ B cells and CD138+ B cells in intestinal epithelial tissues was detected by flow cytometry. (e) Total DNA methylation level was measured. (f) The expressions of DNMT1, DNMT3a, and DNMT3b were detected. (g) DNMT3a protein level was determined by immunofluorescence staining assay. ^∗^*p* < 0.05, ^∗∗^*p* < 0.01, ^∗∗∗^*p* < 0.001 vs. sham group; scale bar = 50 *μ*m.

To determine the severity of intestinal epithelial barrier dysfunction, the expressions of zonula occludens-1 (ZO-1) and occludin were detected. According to the immunofluorescence staining results, the levels of ZO-1 and occludin were significantly reduced after DSS treatment (Figure 2(a)). Similarly, the western blot results showed that the protein levels of ZO-1 and occludin were decreased in the DSS group compared with the sham group (Figure 2(b)). The concentrations of inflammatory cytokines in the serum were measured, and we discovered that IL-10 and TGF-*β* levels were reduced, and IL-6 and TNF-*α* levels were elevated (Figure 2(c)). The flow cytometry result revealed that the number of CD1d+ B cells in intestinal epithelial tissues of DSS group was reduced, while the number of CD138+ B cells was increased (Figure 2(d)). Additionally, DSS treatment remarkably induced DNA methylation level (Figure 2(e)). Based on this result, we further detect the DNA methylation transferase levels containing DNMT1, DNMT3a, and DNMT3b. The result conveyed that the mRNA level of DNMT3a was upregulated the most among the three transferases (Figure 2(f)). We then assessed the DNMT3a level in intestinal epithelial tissues, and the result showed that DSS significantly induced DNMT3a protein level upregulation (Figure 2(g)). These data collectively suggested that the intestinal epithelial barrier dysfunction and DNMT3a level were induced in DSS-induced colitis.

### 3.3. DNMT3a Regulates Intestinal Epithelial Barrier Function and B Cell Differentiation

**Figure 3:**
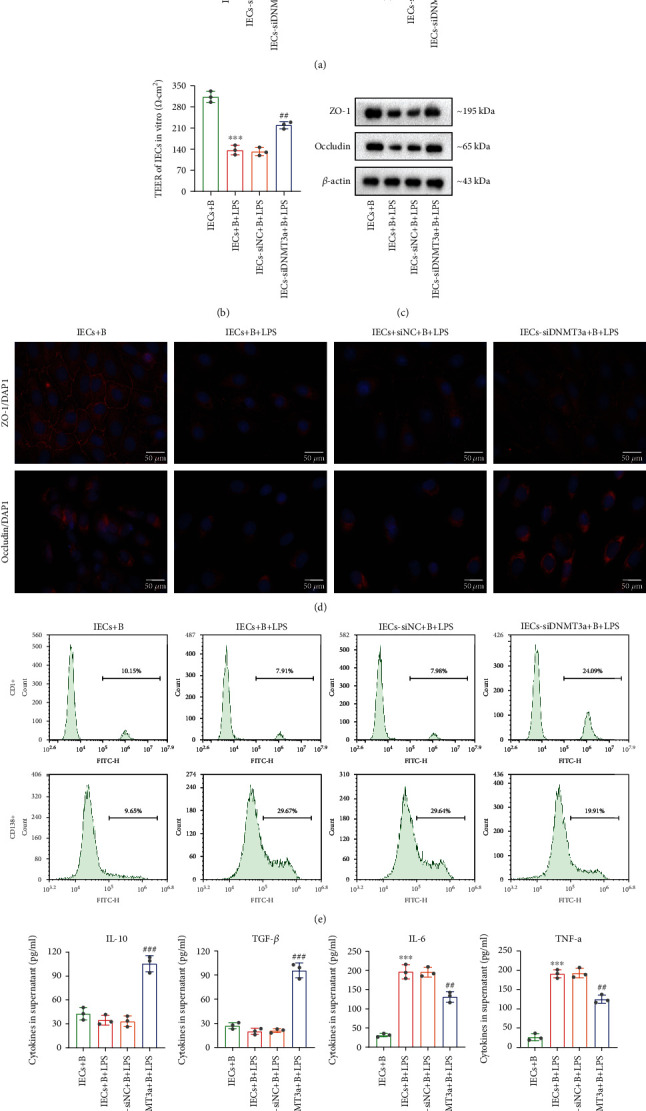
DNMT3a regulates intestinal epithelial barrier function and differentiation of B cells. The primary IECs were isolated from intestinal epithelial tissues of mice. B cells were isolated from the spleens of wide-type mice. Then, the si-DNMT3a or si-NC was transfected into IECs and then treated with LPS (1 *μ*g/mL) for 48 h. Cells were divided into 4 groups: IECs, IECs+LPS, IECs-si-NC+LPS, and IEC-si-DNMT3a+LPS. (a) DNMT3a mRNA level and protein level in each group of IECs were measured. (b) After transfection of si-DNMT3a or si-NC for 24 h, IECs were cocultured with B cells in a cocultural system, and LPS (1 *μ*g/mL) was added into IECs and cultured for 48 h. Four groups were assigned as follows: IECs+B, IECs+B+LPS, IEC-si-NC+B+LPS, and IEC-si-DNMT3a+B+LPS. TEER of IECs in each group was detected. (c) and (d) ZO-1 and occludin protein levels were measured by western blot and immunofluorescence staining. (e) The CD1d+ B cells and CD138+ B cells were detected by flow cytometry. (f) IL-10, TGF-*β*, IL-6, and TNF-*α* levels in cell supernatants were examined. ^∗∗∗^*p* < 0.001 vs. IECs or IECs+B group; ^##^*p* < 0.01, ^###^*p* < 0.001 vs. IEC-si-NC+LPS or IEC-si-NC+B+LPS group; scale bar = 50 *μ*m.

To investigate the role of DNMT3a in regulation of intestinal epithelial barrier function and B cell differentiation, the mouse primary IECs were isolated. Firstly, the isolated IECs were identified, and the results showed that the living cells account for 85% (Supplementary Figure 1(A)) and the anti-CD326 positive cells account for 88% (Supplementary Figure 1(B)), indicating that the isolated cells were largely living IECs. Next, the si-DNMT3a or si-NC was transfected into IECs and then treated with LPS to induce the inflammatory model *in vitro*. The qRT-PCR result showed that DNMT3a mRNA level was increased when treated with LPS, while it was downregulated after si-DNMT3a transfection, indicating that LPS treatment and the transfection were effective (Figure 3(a)). Meanwhile, LPS induced upregulation of DNMT3a protein level, while si-DNMT3a transfection downregulated it (Figure 3(a)). Next, the IECs were cocultured with B cells in a cocultural system, and then LPS was added into IECs to mimic the *in vitro* inflammatory model. We found that LPS induced downregulation of TEER, whereas DNMT3a silencing abolished the effect (Figure 3(b)). Similarly, according to the western blot and immunofluorescence staining results, ZO-1 and occludin protein levels were decreased when treated with LPS, while si-DNMT3a transfection reversed these results (Figures 3(c) and 3(d)). The LPS treatment reduced the number of CD1d+ B cells and increased the number of CD138+ B cells, while si-DNMT3a transfection partly abrogated the effect of LPS (Figure 3(e)). Likewise, IL-10 and TGF-*β* levels were reduced in the supernatants of LPS-induced IECs, while IL-6 and TNF-*α* levels were elevated. However, the DNMT3a silencing reversed these levels (Figure 3(f)). Collectively, these findings indicated that inhibition of DNMT3a alleviated intestinal epithelial barrier dysfunction and induced anti-inflammatory B cell (CD1d+) differentiation induced by LPS.

### 3.4. Overexpression of TNFSF13 in IECs Induces B Cell Differentiation via TACI

**Figure 4:**
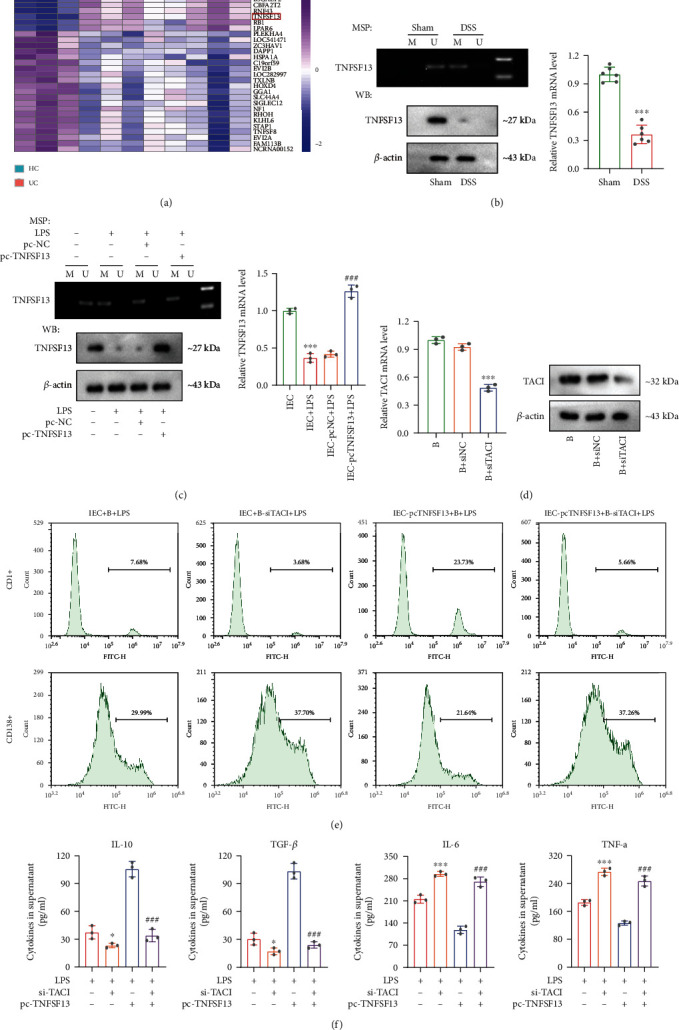
Overexpression of TNFSF13 in IECs induces B cell differentiation via TACI. (a) GSE81211 dataset was used to analyze the genes of high DNA methylation levels in UC, and the screened genes were displayed in heatmap. (b) The intestinal epithelial tissues from mice in the sham and DSS group were collected. TNFSF13 DNA methylation level, mRNA level, and protein level were evaluated. (c) IECs were transfected with pc-TNFSF13 or pc-NC following the treatment of LPS (1 *μ*g/mL) for 48 h. Cells were allocated into 4 groups: IECs, IECs+LPS, IECs-pc-NC+LPS, and IECs-pc-TNFSF13+LPS. The methylation level and mRNA level in IECs and protein level of TNFSF13 in the supernatants were examined. (d) The si-TACI or si-NC was transfected into B cells, and the TACI mRNA and protein levels were detected. (e) Next, IECs with or without pc-TNFSF13 transfection were cocultured with B cells with or without si-TACI transfection. The divided groups were as follows: IECs+B+LPS (TACI was normally expressed in B cells), IECs+B-si-TACI+LPS (TACI was knockdown in B cells), IEC-pc-TNFSF13+B+LPS (TACI was normal expressed in B cells, and TNFSF13 was overexpressed in IECs), and IEC-pc-TNFSF13+B-si-TACI+LPS (TACI was knockdown in B cells, and TNFSF13 was overexpressed in IECs) groups. The numbers of CD1d+ B cells and CD138+ B cells were measured by flow cytometry. (f) IL-10, TGF-*β*, IL-6, and TNF-*α* levels in cell supernatants were examined. ^∗^*p* < 0.05, ^∗∗∗^*p* < 0.001 vs. sham or IEC or B+si-NC or LPS group; ^###^*p* < 0.001 vs. IECs-pcNC+LPS or LPS+si-TACI group.

We next investigate whether TNFSF13 regulates IEC that induced the differentiation of B cells. The GSE81211 dataset was used to screen the genes with high DNA methylation levels in ulcerative colitis, and the screened genes were displayed in a heatmap (Figure 4(a)). According to the previous studies, TNFSF13 is a member of the tumor necrosis factor ligand family, which is secreted by intestinal epithelial cells and plays an important role in the differentiation of immune cells, such as B cells [25, 34]. Our results showed that TNFSF13 DNA methylation level was elevated in intestinal epithelial tissues of DSS-induced colitis, while the mRNA level and protein level were decreased (Figure 4(b)). Next, TNFSF13 was overexpressed in IECs by pc-TNFSF13 transfection and then cocultured with B cells. We discovered that overexpression of TNFSF13 significantly increased the mRNA and protein level of TNFSF13, whereas had no significant effect on TNFSF13 DNA methylation level (Figure 4(c) and Supplementary Figure 1(C)). Transmembrane activator and calcium-modulator and cyclophilin ligand (TACI), expressed in B cells, is a receptor of TNFSF13 [35]. We subsequently knockdown of TACI in B cells by si-TACI transfection, and the result showed that the mRNA and protein level was remarkably reduced (Figure 4(d)). The further result showed that TACI silencing in B cells reduced the number of CD1d+ B cells and increased the number of CD138+ B cells, and overexpression of TNFSF13 in IECs failed to reverse this result (Figure 4(e)). In addition, the si-TACI transfection induced IL-10 and TGF-*β* level downregulation in cell supernatants, while induced upregulation of IL-6 and TNF-*α* levels. Likely,the transfection of pc-TNFSF13 in IECs failed to reverse the levels of these cytokines (Figure 4(f)). Taken together, these data revealed that overexpression of TNFSF13 in IECs enhanced anti-inflammatory B (CD1d+) cell differentiation through TNFSF13/TACI signaling.

### 3.5. DNMT3a Regulates B Cell Differentiation through TNFSF13-Mediated DNA Methylation

**Figure 5:**
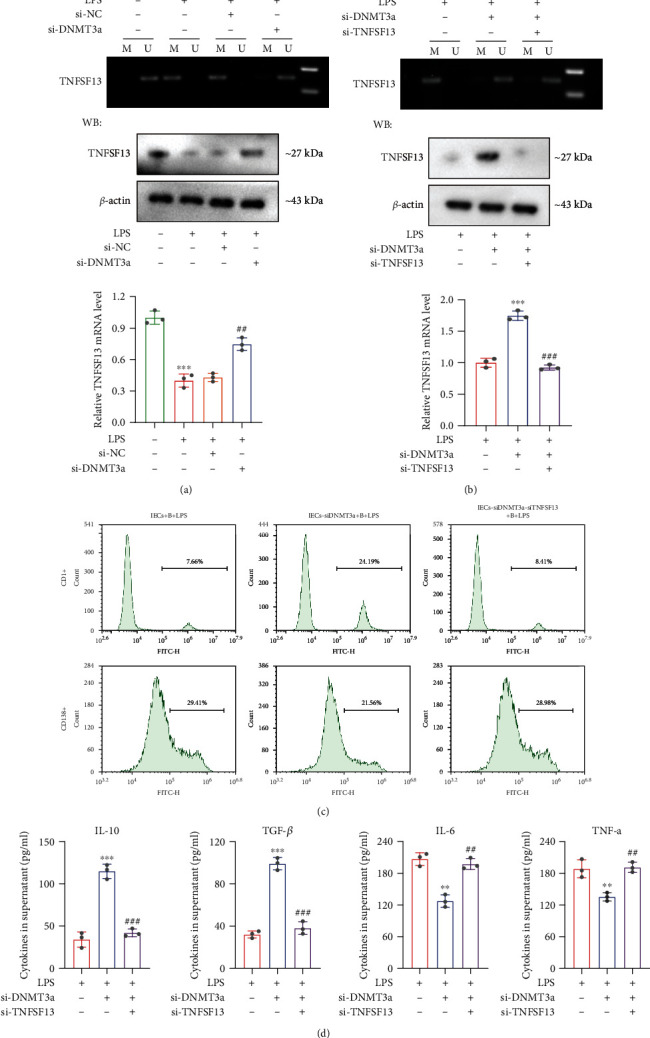
DNMT3a regulates IEC-induced B cell differentiation through TNFSF13-mediated DNA methylation. (a) The si-DNMT3a or si-NC was transfected into IECs and then treated with LPS (1 *μ*g/mL) for 48 h. Cells were grouped into 4 groups: IECs, IECs+LPS, IECs-si-NC+LPS, and IEC-si-DNMT3a+LPS groups. The methylation level and mRNA level in IECs and protein level of TNFSF13 in the supernatants were measured. (b) The si-DNMT3a and si-TNFSF13 were cotransfected into IECs and then treated with LPS (1 *μ*g/mL) for 48 h. IECs were grouped as follows: IECs+LPS, IEC-si-DNMT3a+LPS, and IEC-si-DNMT3a-si-TNFSF13+LPS groups. The methylation level and mRNA level in IECs and protein level of TNFSF13 in the supernatants were measured. (c) IECs were cotransfected with si-DNMT3a and si-TNFSF13 and then cocultured with B cells. IECs were treated with LPS (1 *μ*g/mL) for 48 h. The cocultural cells were assigned into 3 groups: IECs+B+LPS, IEC-si-DNMT3a+B+LPS, and IEC-si-DNMT3a-si-TNFSF13+B+LPS. Flow cytometry assay was conducted to detect the numbers of CD1d+ B cells and CD138+ B cells. (d) The inflammatory cytokines in cell supernatants were measured by ELISA. ^∗∗^*p* < 0.01, ^∗∗∗^*p* < 0.001 vs. Blank or LPS group. Blank: IECs without any treatment; ^##^*p* < 0.01, ^###^*p* < 0.001 vs. LPS+si-NC or LPS+si-DNMT3a group.

To understand whether DNMT3a regulates B cell differentiation through methylation of TNFSF13, si-DNMT3a was transfected into IECs and the TNFSF13 methylation level was measured. We observed that DNMT3a silencing reduced the TNFSF13 methylation level but enhanced the TNFSF13 mRNA and protein level (Figure 5(a)). Next, the si-DNMT3a and si-TNFSF13 were cotransfected into IECs, and we discovered that DNMT3a silencing significantly decreased TNFSF13 methylation level but increased TNFSF13 mRNA and protein level. However,the knockdown of TNFSF13 reversed the data of TNFSF13 mRNA and protein level after DNMT3a silencing but had no significance influence on TNFSF13 methylation level (Figure 5(b)). Meanwhile, DNMT3a silencing enhanced CD1d+ B cell differentiation but reduced CD138+ B cell differentiation, whereas the effect induced by DNMT3a silencing was reversed by knockdown of TNFSF13 (Figure 5(c)). The DNMT3a silencing also inhibited the production of IL-6 and TNF-*α* in cell supernatants while enhancing the release of IL-10 and TGF-*β*. Likely, the cotransfection of si-DNMT3a and si- TNFSF13 negated these results (Figure 5(d)). In addition, we added the experiments with transfection of si-DNMT3a or si-NC in IECs without LPS treatment. According to the results of qRT-PCR and western blotting, DNMT3a expression was significantly reduced, suggesting the effective transfections (Supplementary Figure 1(D)-1(E)). The MSP detection result revealed that the knockdown of DNMT3a had no significant effect on TNFSF13 methylation level (Supplementary Figure 1(F)). In the following, the IECs with si-DNMT3a or si-NC transfection were cocultured with B cells. We found that the knockdown of DNMT3a exerted no significant influence on tight junction protein (ZO-1 and occludin) levels and inflammatory cytokine (IL-10, TGF-*β*, IL-6, and TNF-*α*) levels in B cell supernatants (Supplementary Figure 1(G)-1(H)), implying that knockdown of DNMT3a had no significant effect on TNFSF13 methylation without LPS treatment. Above all, these findings indicated that inhibition of DNMT3a enhanced IEC-induced anti-inflammatory B (CD1d+) cell differentiation through TNFSF13-mediated DNA methylation in UC model.

## 4. Discussion

In the present study, we explored the effect and underlying mechanism of DNMT3a in colitis. We firstly discovered obvious clinical symptoms, enterocyte barrier dysfunction, and pathological damage in mice with DSS-induced colitis. The *in vitro* results revealed that DNMT3a silencing or TNFSF13 overexpression partly abolished the effect of DSS on enterocyte barrier function and B cell differentiation, while si-TACI transfection reversed the effect of TNFSF13 overexpression. DNMT3a silencing enhanced TNFSF13 expression and induced CD1d+ B cell differentiation, whereas these results were reversed after si-TNFSF13 transfection. Our findings deepened the understanding of DNMT3a-mediated methylation on B cell differentiation in UC through the TNFSF13/TACI pathway.

DSS is a type of water-soluble sulfated polysaccharide with antihemorrhagic and anticoagulative effects [36]. The DSS-induced mouse model of colitis has been widely used in basic research to understand the etiology and pathogenesis due to their simplicity, stability, reproducibility, and controllability [37]. In this study, DSS-induced mice showed significant body weight loss, decreased colon length, and obvious histopathological damage, indicating that the animal model was successfully established based on the previous study [38]. The intestinal epithelial barrier damage and abnormal inflammatory response are the common characteristics of DSS-induced colitis [39, 40]. And that, the TJ protein ZO-1 and occludin levels are necessary for maintaining the intestinal mucosal barrier function and attenuating UC [41, 42]. Our study revealed that DSS-induced colitis led to the decline of ZO-1 and occludin levels, anti-inflammatory cytokine levels, and number of CD1d+ B cells, while increased the proinflammatory cytokine levels, number of CD138+ B cells, and DNA methylation level, which were consistent with the previous studies [43–45].

DNA methylation is an important and conserved epigenetic marker associated with genomic stability, inactive transcription, and environmental responses [46, 47]. A number of studies have shown that DNA methylation exert predictive and targeted therapeutic effects in a variety of diseases, including UC [48–50]. A sequencing analysis of peripheral blood mononuclear cell DNA from ulcerative colitis patients suggested that DNMT3a was involved in the regulation of UC [51], which has not been identified in UC patients or models. TNFSF13 has been identified as a critical regulator of B cell development and differentiation, and TNFSF13 can be released from IECs [26, 52], whereas, whether TNFSF13 affects the interaction of IECs and B cell differentiation in UC is unknown. In this study, we found that DSS treatment induced upregulation of DNA methylation level, DNMT3a level, and TNFSF13 methylation level in colon tissue. It has been confirmed that DNA methylation is involved in the regulation of cell differentiation [53]. The *in vitro* results showed that DNMT3a silencing in IECs decreased TNFSF13 methylation level and promoted the CD1d+ B cell differentiation under LPS subjection, and the knockdown of TNFSF13 reversedCD1d+ B cell differentiation but had no significant effect on TNFSF13 methylation level. Meanwhile, we explored the effect of DNMT3a silencing on TNFSF13 methylation and inflammatory response in the absence of LPS treatment. Different to the above data, we observed that DNMT3a silencing exerted no significant effect on TNFSF13 methylation, tight junction protein levels, and inflammatory cytokine levels. The probable reason is that the basal of DNMT3a is really low, and its knockdown without LPS stimulation has little effect on TNFSF13 methylation regulation. TACI is a receptor of TNFSF13 and expressed in B cells [35]. After TACI silencing in B cells, the proinflammatory CD138+ B cell differentiation was enhanced, which was not affected by TNFSF13 overexpression. These data indicated that DNMT3a induced enterocyte barrier dysfunction to exacerbate UC progression via the TNFSF13/TACI-mediated interaction of enterocyte and B cells. To our knowledge, this study for the first time emphasizes the role of DNMT3a in regulating the IEC barrier function and the interaction between IECs and B cells. In addition, we further revealed the mechanism that DNMT3a mediated the communication between IECs and B cells via affecting the TNFSF13 methylation. Similar to our data, Fujii et al. demonstrated that another DNA methyltransferase DNMT1 expression was increased in rectal epithelium of UC patients and involved in the regulation of UC progression [54]. Uzzan *et al.* clarified that TACI was a type III membrane protein with the ability to bind TNFSF1, and TNFSF1 regulated B cell proliferation and differentiation via binding to TACI receptor in inflammatory bowel diseases [55]. It should be noted that these provided evidence may support our data to some extent.

In conclusion, our data showed that DNMT3a was upregulated in DSS-induced colitis mice along with the increased DNA methylation level and proinflammatory cytokines in the colon. In addition, our research provides the evidence that DNMT3a silencing protect against inflammatory through TNFSF13/TACI signaling pathway for enhancing anti-inflammatory CD1d+ B cell differentiation and inhibiting the proinflammatory response *in vitro*. Our study may offer a novel insight for the investigation of UC. Nevertheless, the study is preliminary, and further studies are warranted for deeper discoveries.

## Data Availability

The data used to support the findings of this study are available from the corresponding author upon request.

## Supplementary Materials

Supplementary Materials Supplementary Figure 1: Identifications of the isolated IECs, efficiency of pc-TNFSF13 transfection, and the effect of DNMT3a silencing in LPS-induced inflammatory model. (A) The isolated IECs were stained with the propidium iodide ReadyProbes™ reagent, and the fluorescence intensity was measured by flow cytometry to assess the rate of living IECs. (B) The anti-CD326 antibody was incubated with IECs, and the positive rate was evaluated by flow cytometry. (C) The pc-TNFSF13 or pcNC with fluorescence-labeled EGF was transfected into IECs, and transfection's efficiency was detected by flow cytometry. (D) The si-DNMT3a or si-NC was transfected into IECs, and the DNMT3a mRNA level, DNMT3a protein level (E), and TNFSF13 methylation level (F) were measured. (G) After transfection of si-DNMT3a or si-NC, IECs were cocultured with B cells, the tight junction protein levels (ZO-1 and occludin), and inflammatory cytokine levels (IL-10, TGF-*β*, IL-6, and TNF-*α*) in B cell supernatants were measured. ^##^*p* < 0.01 vs. IEC-siNC group. Supplementary Table 1: primer sequence for RT-qPCR.
