# Supplementary material for: DNMT3a-Mediated Enterocyte Barrier Dysfunction Contributes to Ulcerative Colitis via Facilitating the Interaction of Enterocytes and B Cells
Source: Mediators Inflamm. 2022 May 6;2022:4862763. doi: 10.1155/2022/4862763 (PMC9106515; doi:10.1155/2022/4862763)
Supplement: Supplementary Materials — Supplementary Figure 1: Identifications of the isolated IECs, efficiency of pc-TNFSF13 transfection, and the effect of DNMT3a silencing in LPS-induced inflammatory model. (A) The isolated IECs were stained with the propidium iodide ReadyProbes™ reagent, and the fluorescence intensity was measured by flow cytometry to assess the rate of living IECs. (B) The anti-CD326 antibody was incubated with IECs, and the positive rate was evaluated by flow cytometry. (C) The pc-TNFSF13 or pcNC with fluorescence-labeled EGF was transfected into IECs, and transfection's efficiency was detected by flow cytometry. (D) The si-DNMT3a or si-NC was transfected into IECs, and the DNMT3a mRNA level, DNMT3a protein level (E), and TNFSF13 methylation level (F) were measured. (G) After transfection of si-DNMT3a or si-NC, IECs were cocultured with B cells, the tight junction protein levels (ZO-1 and occludin), and inflammatory cytokine levels (IL-10, TGF-β, IL-6, and TNF-α) in B cell supernatants were measured. ##p < 0.01 vs. IEC-siNC group. Supplementary Table 1: primer sequence for RT-qPCR. [file 4862763.f1.zip › Supplementary table 1.docx]

**Supplementary Table 1.** Primer sequence for RT-qPCR:

| Gene names | primers |
| --- | --- |
| Mus DNMT1 | Forward: GGACCATATCTGCAAGGACATGA  Reverse: ACTGTAGCTTATGGGCTATGACG |
| Mus DNMT3a | Forward: TTGCCATGACAGTGATGAAAGTG  Reverse: TTCTTCTCTTCTTCTGGTGGCTC |
| Mus DNMT3b | Forward: AAGCAACCAGAGAACAAAAGTCG  Reverse: TTCCCGCCATAGCTATTTGTCTT |
| Mus TNFSF13 | Forward: CAACTCTTCAAGTACCCTCTGCT  Reverse: CGGAAGTGTATAGGAGAATGGGG |
| Mus TACI | Forward:TCAAGGAAATCCTGTGTCTCCTG  Reverse: CCTTGCTCTTTTCGGCAATTGAT |
| Mus β-actin | Forward: GCACCGCAAATGCTTCTA  Reverse: GGTCTTTACGGATGTCAACG |
