# Supplementary figures and images for: DNMT3a-Mediated Enterocyte Barrier Dysfunction Contributes to Ulcerative Colitis via Facilitating the Interaction of Enterocytes and B Cells
Source: Mediators Inflamm. 2022 May 6;2022:4862763. doi: 10.1155/2022/4862763 (PMC9106515; doi:10.1155/2022/4862763)

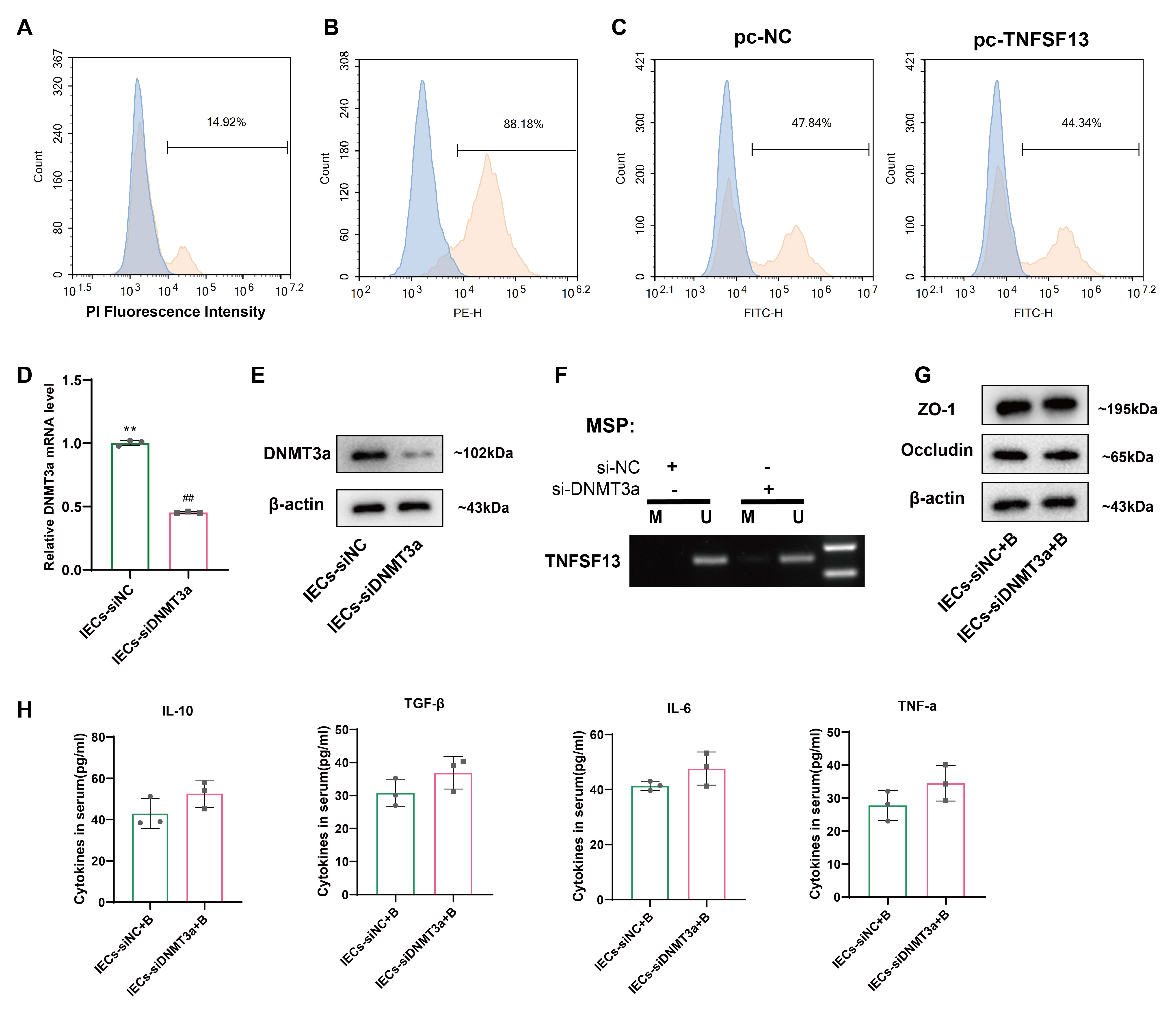

Supplement: Supplementary Materials — Supplementary Figure 1: Identifications of the isolated IECs, efficiency of pc-TNFSF13 transfection, and the effect of DNMT3a silencing in LPS-induced inflammatory model. (A) The isolated IECs were stained with the propidium iodide ReadyProbes™ reagent, and the fluorescence intensity was measured by flow cytometry to assess the rate of living IECs. (B) The anti-CD326 antibody was incubated with IECs, and the positive rate was evaluated by flow cytometry. (C) The pc-TNFSF13 or pcNC with fluorescence-labeled EGF was transfected into IECs, and transfection's efficiency was detected by flow cytometry. (D) The si-DNMT3a or si-NC was transfected into IECs, and the DNMT3a mRNA level, DNMT3a protein level (E), and TNFSF13 methylation level (F) were measured. (G) After transfection of si-DNMT3a or si-NC, IECs were cocultured with B cells, the tight junction protein levels (ZO-1 and occludin), and inflammatory cytokine levels (IL-10, TGF-β, IL-6, and TNF-α) in B cell supernatants were measured. ##p < 0.01 vs. IEC-siNC group. Supplementary Table 1: primer sequence for RT-qPCR. [file 4862763.f1.zip › Supplementary Figure 1-proof.jpg]
